# Enhancing visible light photocatalytic activity of holmium doped g-C_3_N_4_ and DFT theoretical insights

**DOI:** 10.1007/s11356-024-34140-w

**Published:** 2024-07-02

**Authors:** Adem Yavuz, Didem Aydin, Besime Disli, Teoman Ozturk, Berna Gul, Ilkay Hilal Gubbuk, Mustafa Ersoz

**Affiliations:** 1 Center for Materials Research, Integrated Research Centers, Izmir Institute of Technology, Urla, Izmir 35430 Turkey; 2 Department of Chemistry, Faculty of Science, Selcuk University, 42130 Konya, Turkey; 3 Department of Physics, Faculty of Science, Selcuk University, 42130 Konya, Turkey; 4 Advanced Technology Research and Application Center, Selcuk University, 42130 Konya, Turkey

**Keywords:** Holmium doping, g-C_3_N_4_, Methylene Blue, Visible light photocatalysis, DFT calculations

## Abstract

In the search of novel photocatalysts to increase the effect of visible light in photocatalysis, g-C_3_N_4_ (CN) has become a shining star. Rare earth metals have been used as dopant material to reinforce the photocatalytic activity of CN due to their unique electron configuration recently. In this present study, the pure and different amounts of Ho-doped g-C_3_N_4_ (HoCN) photocatalysts were successfully synthesized using urea as a precursor by the one-pot method. Morphological, structural, optical, and vibrational properties of the synthesized photocatalysts were characterized by SEM, EDX, XRD, TGA, XPS, FTIR, PL, TRPL, Raman, DRS, and BET analyses. In addition, theoretical calculations using density functional theory (DFT) were meticulously carried out to delve the changes in the structural and electronic structure of CN with holmium doping. According to calculations, the chemical potential, electrophilicity, and chemical softness are higher for HoCN, while HOMO–LUMO gap, dipole moment, and the chemical hardness are lower for the pure one. Thus, holmium doping becomes desirable with low chemical hardness which indicates more effectivity and smaller HOMO–LUMO gap designate high chemical reactivity. To determine the photocatalytic efficiency of the pure and doped CN photocatalysts, the degradation of methylene blue (MB) was monitored under visible light. The results indicate that holmium doping has improved the photocatalytic activities of CN samples. Most strikingly, this improvement is noticeable for the 0.2 mmol doped CN sample that showed two times better photocatalytic activity than the pure one.

## Introduction

In the recent past, the balance of nature has been disrupted, and environmental problems that threaten the lives of living things on earth have been increasing due to growing population, industrialization, and energy consumption. As a consequence of these negative conditions, water sources are rapidly becoming polluted. Moreover, pollution entering water, such as pesticides, herbicides, antibiotics, and industrial wastes, seriously affects human health (Abbasi_Asl et al. 2023; Ambaye et al. 2021; Gothwal and Shashidhar 2015; Tang et al. 2021). Among the industrial wastes, textile dyes have mutagenic and carcinogenic properties and are closely associated with environmental contamination and a large number of diseases in living things (Singh and Chadha 2016). It is estimated that hundreds of thousands of tons of synthetic dyes are produced worldwide every year, and more than 15% of these toxic products are released into the environment as industrial waste (Nazri and Sapawe 2020). Therefore, developing efficient wastewater treatment methods plays a crucial role in the environment and human health.

To purify water from pollutants, some techniques can be applied, such as filtering, advanced oxidation, biodegradation, and photocatalysis (Bulai and Venturino 2016; dos Santos et al. 2007; Lu et al. 2022). Among them, photocatalysis is a cheap, environmentally friendly, and efficient technique that does not generate secondary waste and has promising applications in recent years. Since Fujishima and Honda’s seminal work on water splitting by photocatalysis (Fujishima and Honda 1972), many photocatalysts have been widely studied. Most semiconductor photocatalysts can be excited with high energy ultraviolet (UV) light due to their wide band gaps. While only 3–5% of the sunlight hitting the Earth’s surface is UV light, around 40% of the solar radiation constitutes visible light (Frederick et al. 1989; Wang and Yu 2023). Since UV light is limited in photocatalysis, there has been a great struggle so far in the search for materials with photocatalytic activity under visible light and increasing their photocatalytic efficiency.

In search of economical, ecological, and stable visible light semiconductor photocatalysts, a conjugated polymeric semiconductor, graphitic carbon nitride (CN), has fascinated the scientific community and attracted tremendous attention, recently. CN is an n-type semiconductor that is polymeric, stable, nontoxic, metal free, abundant in its elements, and possesses a moderate band gap energy (2.7-2.8 eV). It has widespread use not only in the field of photocatalysis but also in energy conversion and sensor and battery applications. Since the showing of H_2_ production under visible light performed by Wang and colleagues (Wang et al. 2009), CN has found widespread use not only in the field of photocatalysis but also in energy conversion and sensor and battery applications. CN-modified TiO_2_ nanosheets were applied as a photoanode in dye-sensitized solar cells and enhanced the efficiency by approximately 28% (Xu et al. 2015). In a study where CN is incorporated into perovskite layer, 19.49% efficiency was achieved passivating electron–hole recombination and enhancing crystallization. Moreover, the CN incorporation improved the morphology of the perovskite films and caused the growth rate of the perovskite film to decrease (Jiang et al. 2018). In a gas sensing study (Cai et al. 2021), a gas sensor using CN nanobelts was designed for reversible detection of NO_2_. CN nanobelts applied as fluorescent nanoprobes yielded long photoluminescence emission wavelength. Regarding energy conversion, CN has been used in sodium-ion batteries as negative electrode. In a study where CN composited with carbon was used as an anode, sodium storage capacity was improved (Weng et al. 2019). Another interesting use of CN is phototherapy, and an intelligent nanoregulator coated with CN layer was designed by Zhang et al. to fulfill efficient delivery (Zhang et al. 2020b). With this nanoregulator, water molecules were disintegrated catalytically, and tumor hypoxia was alleviated.

Although CN is used extensively because of its marvelous traits, there are some shortcomings, such as the high recombination rate of electron–hole pairs produced by light, low quantum efficiency, low surface-active area (< 10 m^2^/g), poor electrical conductivity and high charge transfer resistance (Xu et al. 2013b). These restrictions were tried to be solved with various approaches such as doping with metals (Ding et al. 2015; Li et al. 2016; Wang et al. 2017, 2019; Zhang et al. 2016b) and non-metals (Fang et al. 2015; Pérez-Torres et al. 2023; Qu et al. 2018; Wang et al. 2015b, 2010) or forming heterojunctions (Guo et al. 2019; Jin et al. 2019; Zhang et al. 2020a). Compared to others, doping is a widespread method for improving the photocatalytic efficiency of materials. Incorporating dopants into the CN structure allows the band gap energy to be narrowed, while the electron–hole recombination rate can be suppressed and the surface area expanded (Jiang et al. 2017; Phoon et al. 2022).

Metal doping is a desirable and attractive engineering to improve the photocatalytic activity of CN (Yan et al. 2019). Among metals, rare earth elements can capture electrons due to their unusual 4f electron configuration and are highly remarkable for increasing the photocatalytic activity of CN (Ismael 2023). The unfilled 4f electron configuration facilitates the capture of the photo-generated electrons and delays the recombination of electron–hole pairs. In addition, f orbitals of rare earth elements constitute complexes with various Lewis base adducts (Parnicka et al. 2017). Thus, rare earth ions settled in CN include oxygen vacancies and surface defects.

In the last decade, lanthanide-derived compounds have drawn attention because of high-efficiency light converting properties based on their electronic, optical, and chemical properties that commonly originate from their 4f electrons (Hafez et al. 2011; Saif and Abdel-Mottaleb 2007; Wang et al. 2011; Xu et al. 2002; Zalas and Klein 2012). One of the first studies using rare earth metals in the photocatalyst of CN was done by Jin et al. (2015), and they examined the photocatalytic activity of cerium (Ce)-doped CN by the degradation of Rhodamine B (RhB). They synthesized the pure and Ce-doped CN using cerium sulfate tetrahydrate and melamine as precursors and investigated the breakdown of RhB under visible light. They concluded that doping reduces the band gap energy and electron–hole recombination ratio and inhibits crystal structure growth. They stated that the highest photocatalytic performance obtained belonged to the Ce (0.5%)-doped material. Xu et al. (2013a) examined the effect of europium (Eu) doping concentration on the photocatalytic performance of CN by observing the degradation of MB solution under visible light irradiation. It was stated that Eu doping increased the photocatalytic activity for all samples, and the highest increase was obtained for the sample with a europium content of 0.38% by weight. Experimental and theoretical characterization results supported the study, and the obtained photocatalytic improvement was attributed to some effects such as increase in electron–hole separation rate, better photo-absorption, and larger specific surface area. In another study on Eu, Wang and co-workers (Wang et al. 2018) synthesized hollow-structured lantern-like Eu-doped CN. By obtaining larger specific surface areas with these hollow structures, Eu-doped CN exhibited better photocatalytic activity around six times with the degradation of RhB and tetracycline. Xu et al. (2014) used erbium (Er) and thulium (Tm) to extend the photocatalytic efficiency of CN, and this extension was verified by the photodegradation using a red laser. Chen et al. improved the photocatalytic activity of O-doped CN by decorating with Gd_2_O_3_ and selected sulfamerazine as the target pollutant (Chen et al. 2021). In another interesting study, Li et al. (2020) synthesized samarium (Sm)-doped CN as a photocatalyst and used it to separate Tylosin, a hard-to-degrade antibiotic species, from water. They stated that doping increases the porosity and load-carrying capacity and decreases the band gap energy. The photocatalytic performance of the 0.025% Sm-doped material was 3.55 times higher than that of the pure CN.

Among the rare earth elements, holmium has drawn attention recently and found a place in many application areas, such as solar cells, photodiodes, batteries, solid-state lasers, and photocatalysis (Duan et al. 2018; Ganesan 2009; Imenkov et al. 2009; Pierre and Preminger 2007; Shi et al. 2009). In this study, Ho-doped CN photocatalysts and its photocatalytic activity were reported for the first time in the literature. Considering the widespread use and unusual properties of Ho, the pure and holmium (Ho)-doped CN photocatalysts were synthesized using urea as a precursor, investigated by theoretically and characterized by structural, optical, and morphological methods. The effect of Ho doping on photocatalytic performance was studied under visible light irradiation through the degradation of MB as a model pollution. By using different amounts of holmium-doped CN nanoparticles (0.1 mmol, 0.2 mmol, 0.3 mmol), the photodegradation rates of MB were determined under visible light, and it was seen that the best result was with the one doped with 0.2 mmol. Moreover, density functional theory (DFT) was used to elucidate atomic array structures and the highest occupied molecular orbital (HOMO) and lowest unoccupied molecular orbital (LUMO) levels. The changes in the properties of CN, such as electron transfer mechanism and absorption and its effects on the photocatalytic performance, were determined in detail through the proposed theoretical model and experimental outcomes.

## Experimental details

### Materials

Urea (CH_4_N_2_O, 99.0–100.5%), Holmium(III) nitrate pentahydrate (Ho(NO_3_)_3_·5H_2_O, 99.9%), and methylene blue (C_16_H_18_ClN_3_S·*x*H_2_O) were purchased from Sigma-Aldrich. All chemicals were of analytical purity. Distilled water was used throughout all washing procedures, and deionized water was utilized in the photocatalytic experiments to obtain aqueous solutions.

### Synthesis of photocatalysts

High-temperature calcination using urea was used as the method for synthesis. CN was prepared based on studies in the literature (Li et al. 2018, 2019). First, 10 g of urea was accurately weighted and added to 50 ml of a porcelain crucible covered with a cap placed in a muffle furnace. The crucible at a 5 °C/min rate was heated to 550 °C in a nitrogen atmosphere. Afterward, it was kept at this temperature for about 3 h. After cooling to room temperature, the yellow powder was cautiously washed by centrifugation at 6000 rpm with distilled water and dried for 12 h at 80 °C. The synthesized sample was labeled as CN.

Ho-doped CN photocatalysts were prepared as follows (Fan et al. 2019). First, 6 g of powder urea and *x* mmol (*x* = 0.1, 0.2, 0.3) of Ho(NO_3_)_3_·5H_2_O was deposited into a ceramic crucible with a volume of 50 ml. The mixtures were stirred with a glass rod, and the lid of the crucible was closed. The mixtures were heated to 135 °C and kept for 15 min to prevent rapid evaporation of the urea. Then, the crucible was placed in a muffle furnace and further heated to 500 °C with a heating rate of 20 °C/min and preserved at 500 °C for 2.5 h under a nitrogen atmosphere. Finally, after cooling to room temperature, the obtained products were washed by centrifugation at 6000 rpm with distilled water and dried overnight at 80 °C. The synthesized products were named as 0.1 HoCN, 0.2 HoCN, and 0.3 HoCN, according to the amount of holmium they contained (0.1, 0.2, 0.3 mmol), and studied as the quantity parameter.

### Characterization

The crystal structure of the photocatalysts was analyzed using the Bruker X-ray diffraction (XRD) system. Fourier transform infrared (FTIR) spectra were recorded with a Perkin Elmer spectrum 100 FT-IR spectrometer (ATR). The morphology of the samples was obtained via Zeiss EVO LS-10 scanning electron microscope (SEM). Raman spectra were recorded with Renishaw InVia Qontor Raman spectrometer, using a 785-nm laser with 100% power on the sample 10-s exposure, and 20 accumulations in the wavenumber region 200–2000 cm^−1^, using × 50 objective lens. Photoluminescence (PL) and time-resolved photoluminescence (TRPL) measurements were performed by using a FS5 Spectrofluorometer (Edinburgh Instruments, UK) at room temperature. The surface area measurements were carried out with Micromeritics (USA) 3Flex Adsorption Analyzer and calculated from the N2 sorption isotherm using the Brunauer–Emmett–Teller (BET) models. The samples were degassed at 120 °C for 3 h. The valid relative pressure range for the specific BET surface area was calculated using a Rouquerol plot. Thermal gravimetric analyses (TGA) were performed using TGA instrument (Perkin Elmer, Diamond) under a nitrogen atmosphere between 25 and 800 °C at a heating rate of 10 °C/min. Reflectance spectra were obtained by a Shimadzu 3600 plus spectrophotometer, where BaSO_4_ was used as a reference. X-ray photoelectron spectroscopy (XPS) measurements were performed by using a Thermo Scientific K-Alpha (Thermo Fisher) spectrometer. A monochromatic Al K Alpha source was used for electron excitation, and a hemispherical electron analyzer was fixed at 45° with respect to the surface normal, and the spot size was 400 μm. The high-resolution spectra of C, N, O, and Ho were recorded with 10 scans. Curve fitting was carried out with XPSPeak analysis software.

### Photocatalytic experiments

Photocatalysis experiments were performed to investigate the photocatalytic activities of the nanoparticles by monitoring the degradation of MB under visible light irradiation at ambient temperature. Firstly, 20 mg of photocatalyst was weighed on a precision balance and added into a reactor containing 50 ml MB dye solution of 10 ppm. Secondly, the mixed solution was stirred continuously for 30 min in the dark to reach an adsorption–desorption equilibrium between the photocatalysts and MB. Then, the reactor was exposed to visible light using 120-W lamps at 30-min intervals. At the end of 30 min, an amount of the mixture solution was taken and centrifuged. The absorbance of the supernatant liquid was measured with a spectrophotometer. The solution was stirred magnetically at room temperature inside the reactor at a moderate level throughout the measurement.

### Computational details

Gaussian 16 has been used for the density functional theory (DFT) calculations (Frisch et al. 2016). All molecular geometries were optimized using the B3LYP exchange–correlation functional (Tirado-Rives and Jorgensen 2008) in conjunction with the People’s split valence 6-31G(d,p) basis set (Binkley et al. 1980; Francl et al. 1982; Gordon et al. 1982; Pritchard et al. 2019; Schuchardt et al. 2007) for CN and Ahlrichs-Karlsruhe def2-TZVP triple zeta valence basis set (Gulde et al. 2012) for Ho. HOMO–LUMO locations and the vibrational calculations were also executed using the same functional and basis sets. The Hirsfeld method was used to calculate the population analysis and dipole moment more accurately. The keyword int = ultrafine was used to improve the accuracy of the calculations. The Raman and IR spectra were calculated using pure Lorentzian band shapes with a full width at half height of 12 cm^−1^. The computed wavenumbers have been scaled by 0.9608 for C, N, and H, by 0.9654 for Ho. The structures and surface plots were visualized using Gaussview 6.0. (Dennington et al. 2016).

## Results and discussions

### Computational results

The dipole moments and global chemical reactivity parameters, electronegativity (χ), chemical potential (µ), chemical hardness (η), chemical softness (S), and electrophilicity index (ω) were obtained from Eqs. (1–5), according to Koopmans’ theorem (Koopmans 1934; Pearson 1988), by correlating the HOMO and LUMO energies to the negative of electron affinity (EA) and ionization potential (IP), respectively. The calculated values are given in Table 2.

1 $$\begin{array}{c}\chi = \frac{IP+EA}{2}\end{array}$$

2 $$\begin{array}{c}\mu = -\chi =-\left(\frac{IP+EA}{2}\right)\end{array}$$

3 $$\begin{array}{c}\eta = \frac{IP-EA}{2}\end{array}$$

4 $$\begin{array}{c}S = \frac{1}{\eta }\end{array}$$

5 $$\begin{array}{c}\omega = \frac{{\mu }^{2}}{2\eta }\end{array}$$

**Fig. 1:**
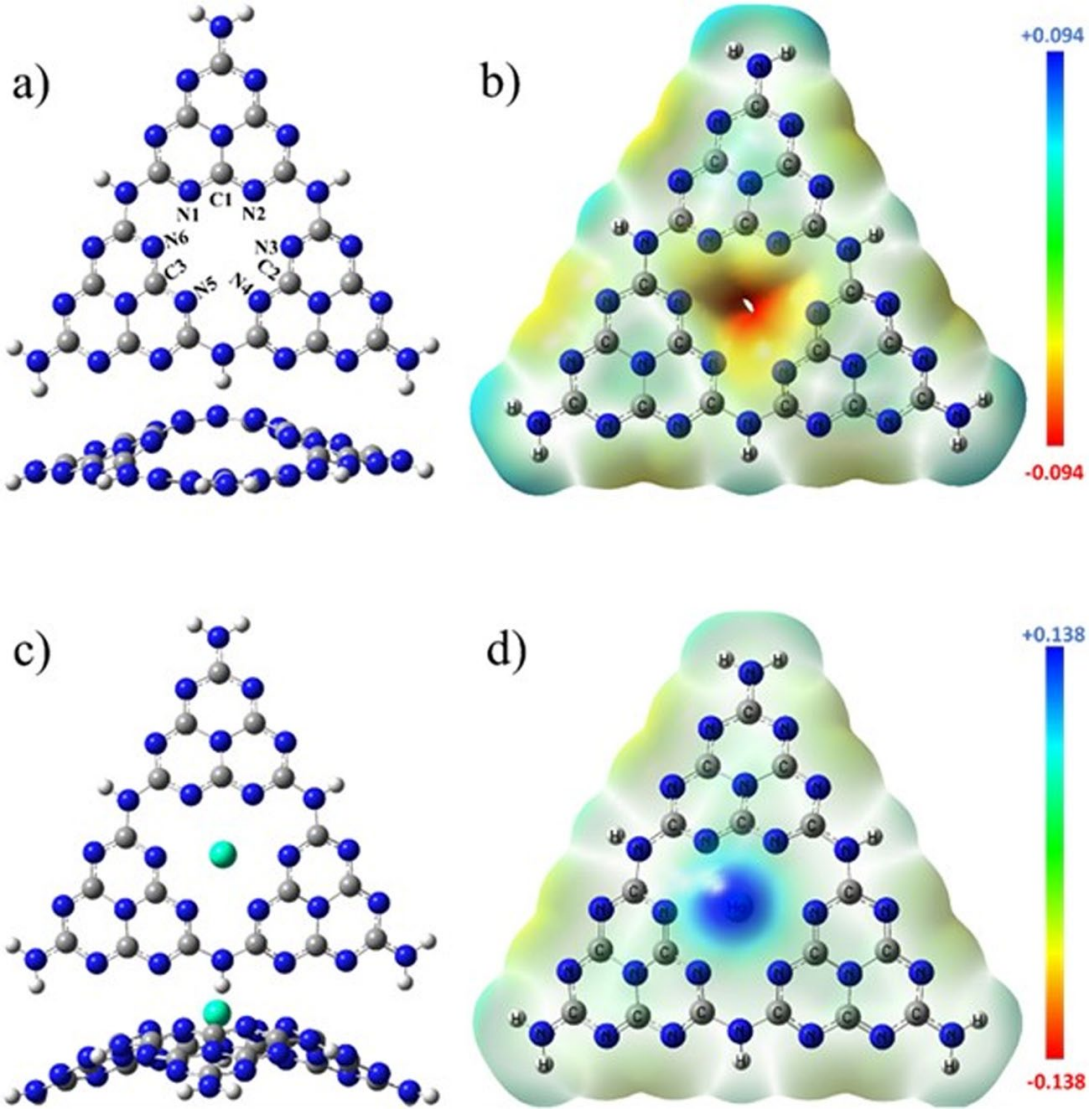
Optimized molecular structure front (up) and side (bottom) views and MEP surfaces of **a**, **b** CN and **c**, **d** HoCN

**Fig. 2:**
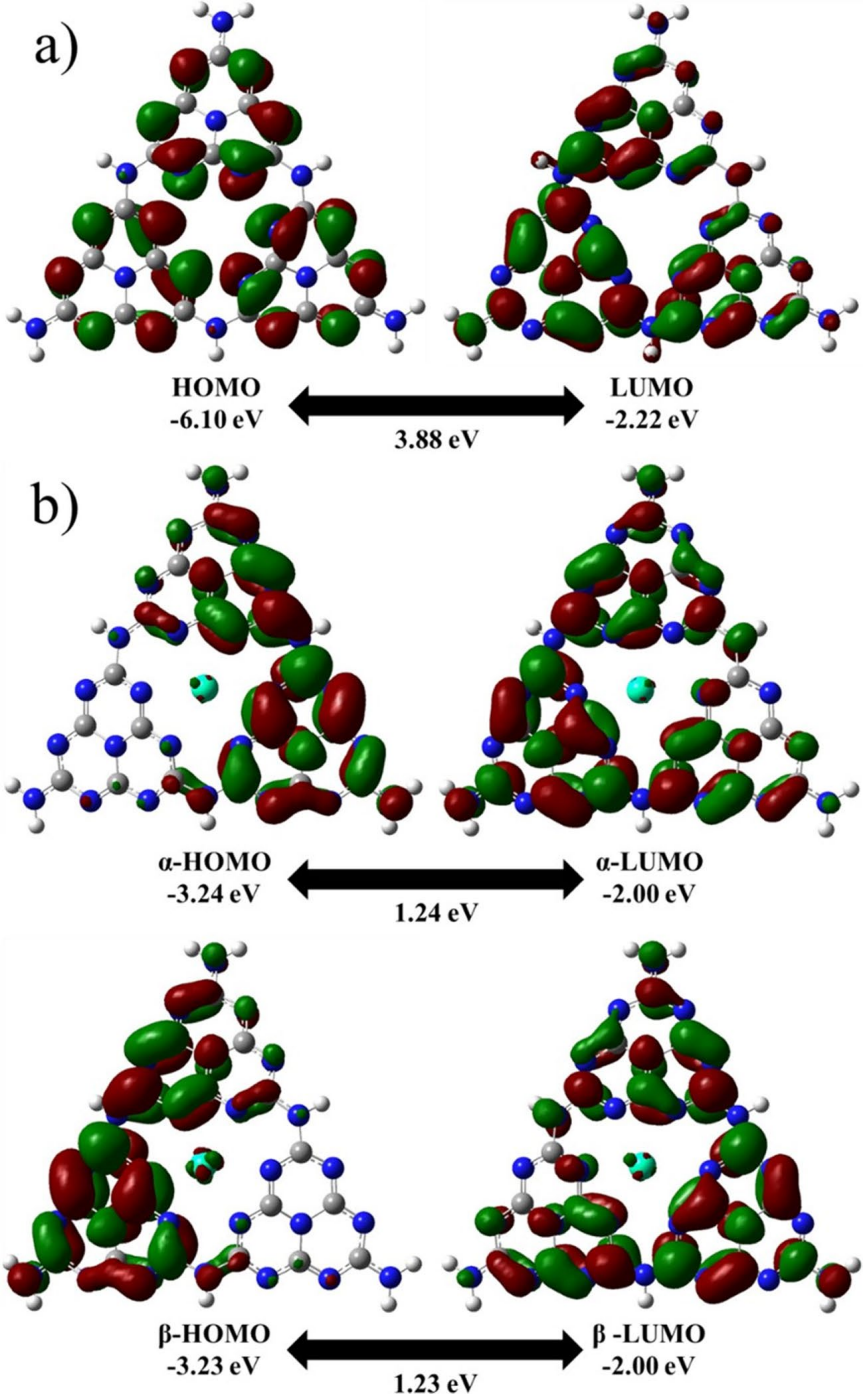
Calculated HOMO–LUMO plots of **a** CN and **b** HoCN

The optimized structure of CN consists of three adjacent tri-s-triazine units, which were oriented at three different planes. These units formed a wave-like structure with a central vacancy, as shown by the front and side view orientations in Fig. 1a (Zhou et al. 2019). The N and C atoms located at the periphery of the vacancy in the center of CN were labeled N1, N2, N3, N4, N5, N6, C1, C2, and C3 in Fig. 1a. As seen in Fig. 2a, the highest occupied molecular orbitals (HOMO) extended over the N atoms located at the periphery of the vacancy. On the other hand, the lowest unoccupied molecular orbitals (LUMO) covered the N and C atoms located at the same periphery. These results indicate that these atoms have a high chemical reactivity; therefore, the HoCN structure was obtained by adding Ho to the central vacancy in CN. The Ho atom was located at the center of the CN, as seen in Fig. 1c. It was not bonded to any other atom but tends to attract the neighboring N atoms. This led to a slight decrease in N–C–N angles and an increase in N–C bond lengths on the order of picometers (see Table 1), giving the HoCN system an umbrella-like geometry (Sarkar et al. 2020). The average distances between Ho-C and Ho-N were found to be 2.96 Å and 2.54 Å, respectively.

**Table 1:** C, N bond lengths and Ho distances with N atoms and N*–*C*–*N angles within the periphery of vacancy of CN

Bond lengths (Å)		
	CN	HoCN
C1-N1	1.32	1.33
C1-N2	1.33	1.33
C2-N3	1.33	1.32
C2-N4	1.32	1.34
C3-N5	1.33	1.34
C3-N6	1.33	1.32
Ho-N1	-	2.54
Ho-N2	-	2.54
Ho-N3	-	2.53
Ho-N4	-	2.55
Ho-N5	-	2.55
Ho-N6	-	2.54
Bond angles (°)		
N1-C1-N2	120.8	117.3
N3-C2-N4	120.8	117.8
N5-C3-N6	121.1	117.8

Adsorption energy (*E*_*ad*_) of Ho towards the pristine CN can be calculated as follows (Umar et al. 2022):

6 $${E}_{ad}= {E}_{\text{complex}}-({E}_{\text{adsorbate}}+{E}_{\text{host}} )$$

where *E*_complex_, *E*_adsorbate_, and *E*_host_ represent the total energies of the HoN, Ho, and CN, respectively. The adsorption energy of Ho is − 4.08 eV, which is a very negative value; this indicates that Ho has exhibited strong interaction with CN.

The HOMO and LUMO distributions of pristine CN and HoCN are shown in Fig. 2, and these are important as they indicate the regions prone to electron withdrawal and acceptance, respectively (Sarkar et al. 2020). The results showed that HOMO (− 6.10 eV) and LUMO (− 2.22 eV) for CN and singly occupied α-HOMO (− 3.24 eV), α-LUMO (− 2.00 eV), β-HOMO (− 3.23 eV), and β-LUMO (− 2.00 eV) for HoCN. When Ho atoms are adsorbed on CN, the mechanism that governs the process is the concurrent tendency to donate and accept electron pairs. This process is reflected in the energy levels, including *E*_HOMO_, *E*_LUMO_, and the corresponding *E*_HOMO_-*E*_LUMO_ energy gap (Δ*E*). The Δ*E* value indicates the ease with which electron pairs can be promoted (Nnadiekwe et al. 2023; Sarkar et al. 2020). In HoCN, both the HOMO and LUMO levels increased, and there was a significant decrease in Δ*E* compared to isolated CN, as shown in Fig. 2 and Table 2. In isolated CN, the HOMO covered only the N atoms, while the LUMO extended to both the N and C atoms. Both the HOMO and LUMO levels extended over almost the entire CN surface. Specifically, upon the addition of Ho, the HOMO shifted from the N atoms to both N and C atoms. The Ho atom occupied only a small portion of both the HOMO and LUMO, while both orbitals extended over almost the entire surface of the complex. Compared to isolated CN, the HoCN complex exhibited a shift of HOMO and LUMO towards more positive energy levels, and a reduction in the HOMO–LUMO gap had been observed. This lower Δ*E* value indicates a higher chemical reactivity of the molecule. Moreover, a low Δ*E* in the ground state suggests that it is more sensitive to UV/visible light. The dipole moment of CN was calculated to be 1.83 D. However, after the addition of Ho to CN, the dipole moment was decreased to 1.02 D. This decrease in the dipole moment is relatively undesirable for the solubility of the complex, particularly in polar solvents like water.

**Table 2:** HOMO, LUMO, HOMO–LUMO gap (Δ*E*), dipole moment, and quantum chemical parameters of CN and HoCN

	*E*_LUMO_	*E*_HOMO_	Δ*E*	Dipole Moment	*χ*	*μ*	*ɳ*	*S*	*ω*
	(eV)			(Debye)	(eV)	(eV)	(eV)	(eV^−1^)	(eV)
CN	− 2.22	− 6.10	3.88	1.83	4.16	− 4.16	1.94	0.52	4.46
HoCN	− 2.00	− 3.24	1.24	1.02	2.62	− 2.62	0.62	1.61	5.54

Molecular electrostatic potential (MEP) surfaces for CN and HoCN are given in Fig. 1b and d, where the red, blue, and green regions on the MEP surface represent the negative, positive, and neutral electrostatic potential (ESP) regions, respectively (Bihain et al. 2022; Kuila et al. 2020; Sarkar et al. 2020). The CN molecule has an area at the center vacancy that attracts electrophiles more easily. In the optimized structure of Ho added into this vacancy of CN, the MEP surface indicates that in the center, it has a positive electrostatic potential while the edges become almost neutral. As a result, the Ho atom in the center becomes more prone to nucleophilic reactions, which indicates the most reactive site.

Chemical potential (*µ*), electrophilicity index (*ω*), and chemical softness (*S*) are higher in HoCN compared to CN, while chemical hardness (*η*) is lower. Chemical hardness (*η*) represents the ability of a molecule to resist electron distortion and contribute non-bonding electrons when interacting with an adsorbent. A high hardness indicates low reactivity, while soft molecules are more effective at adsorption (Asif et al. 2021; Miar et al. 2021; Nnadiekwe et al. 2023; Pearson 1988; Sarkar et al. 2020). Ho-doped CN was found to be softer than CN, as evidenced by a higher softness value and lower hardness. The softness and electrophilicity index values of HoCN were 1.61 eV^−1^ and 5.54 eV in contrast to 0.52 eV^−1^ and 4.46 eV for CN, respectively. The HoCN showed improved chemical reactivity and charge transfer compared to pristine CN due to a significant increase in global softness and electrophilicity parameters (Kuila et al. 2020; Wei et al. 2023). The molecular stability and sorbent properties of the HoCN are estimated via these parameters. After a comprehensive analysis of the data, it has been realized that Ho has the potential to be an effective sorbent for CN. Notably, it exhibited a smaller HOMO–LUMO gap, a higher softness (*σ*), and the electrophilicity index (*ω*), making it a desirable option. Furthermore, the HOMO–LUMO distribution was delocalized, indicating greater potential for visible light photocatalytic use. Consequently, the results suggested that HoCN is the optimal choice for this objective.

### Structural analysis

#### XRD analysis

**Fig. 3:**
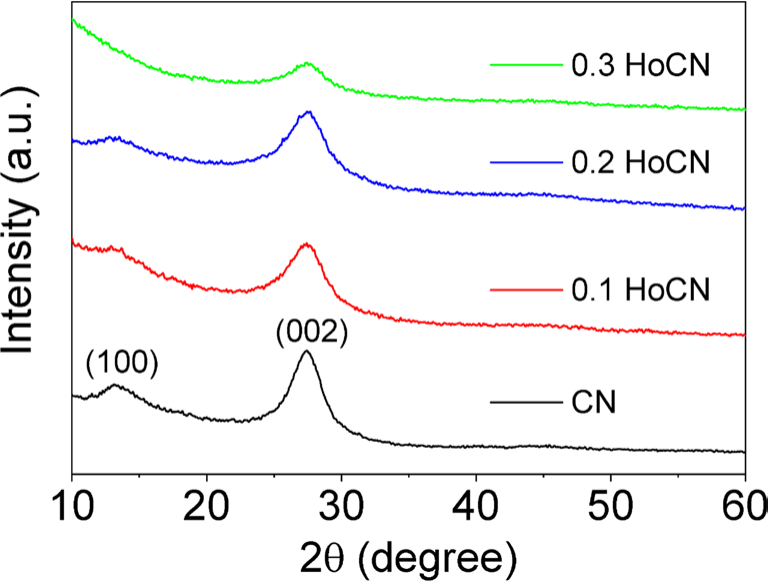
XRD peaks of the nanoparticles

Figure 3 shows the X-ray diffraction (XRD) patterns of the synthesized CN and a series of HoCN photocatalysts. The crystal structure of the four samples was studied by X-ray diffraction with CuKα radiation (*k* = 1.54056 A˚) operated at 45 kV and 40 mA in a range of 10–60°. It is seen that while the peak in the vicinity of 13° is designated to (100) plane with an interlayer distance of 0.667 nm, the peak around 27° is indexed to (002) plane with an interlayer distance of 0.326 nm. The less intense peak at ∼ 13° is thought to be due to in-plane reflections of tri-s-triazine motifs (100), while the peak at ∼ 27° is considered to be from graphite-like interlayer (002) stacking of the aromatic ring. Additionally, no characteristic holmium peak was found in the XRD patterns. This was thought to be because either Ho ions were incorporated into the carbon nitride matrix crystalloid or the holmium was very small and highly dispersed (Shi et al. 2009; Wu and Chen 2004). Interestingly, the peaks belonging to 0.3 mmol Ho-doped sample decrease with increasing Ho amount. The intensity of the peaks indexed to the (100) diffraction plane gradually decreases with the increasing amount of Ho dopant due to the interactions between Ho and CN. On the other hand, when the 0.2 mmol Ho doping is increased to 0.3 mmol, the decrease in the peak of the (002) plane indicates that its crystallinity is impaired and the triazine rings are weakened by the Ho contribution (Deng et al. 2019; Wang et al. 2022).

#### Vibrational properties of CN and HoCNs

**Fig. 4:**
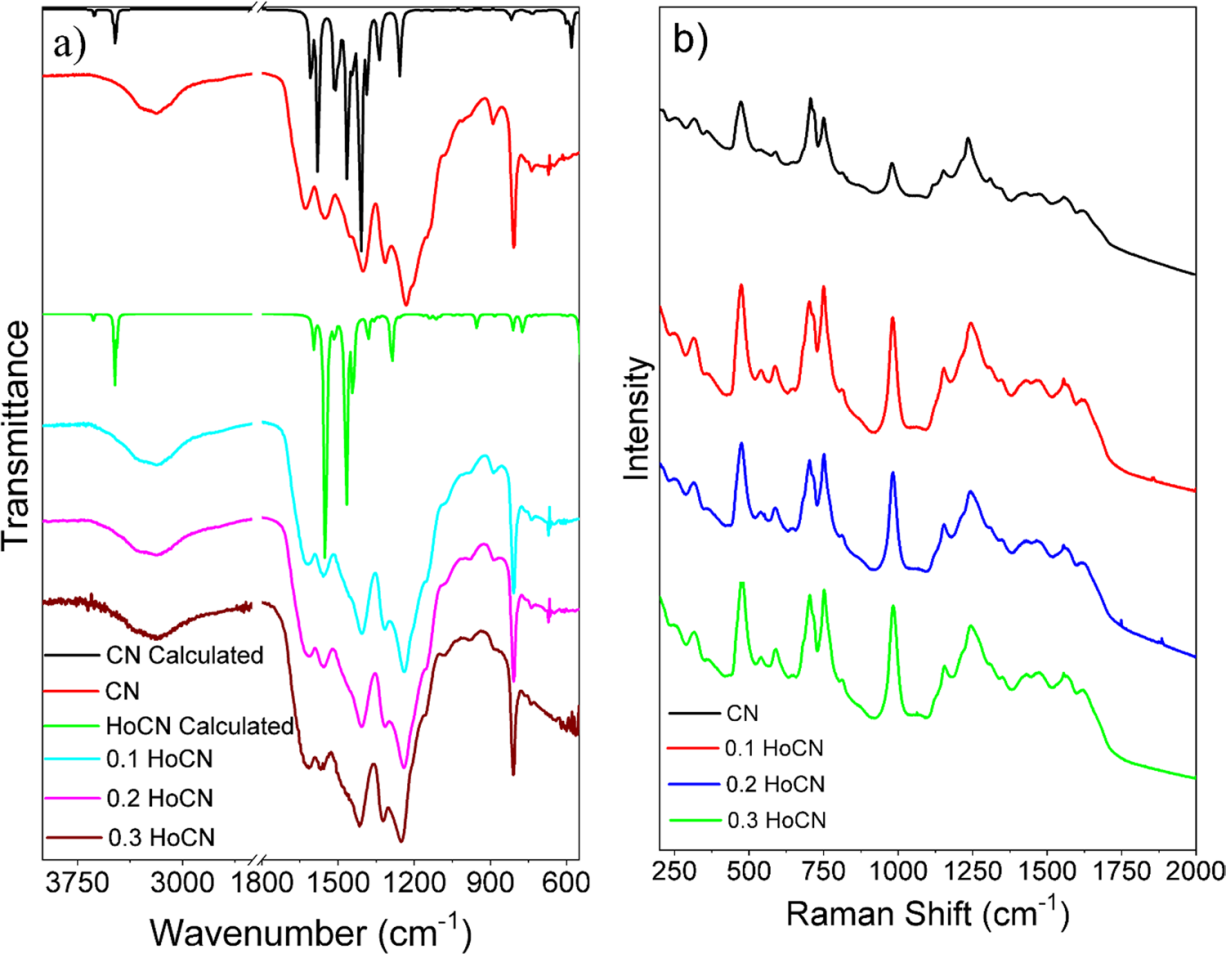
FTIR spectrum of each sample and calculated IR spectrum of isolated CN and HoCN (**a**); raw Raman spectra of CN and HoCN samples (**b**)

This section investigated the vibrational properties of CN and HoCN composites using FTIR, Raman spectroscopic methods, and DFT vibrational calculations. Figure 4a and Table 3 show the experimental FTIR spectra and calculated IR of the prepared pure CN and HoCN materials. The simulated IR spectra of isolated CN and HoCN were compared with the experimental spectrum of each sample, and a good agreement was found. The structural differences between the synthesized samples were determined and explained using FT-IR spectroscopy. The chemical functional groups of CN and the HoCN composite were analyzed using the FT-IR spectra, providing insights into the types of bonds and functional groups in the materials. This analysis can help explain the chemical properties of the samples. The peak attributed to triazine breathing mode and characteristic peak appears at 808 cm^−1^. Absorption peaks seen at 1232, 1313, 1402, 1551, and 1628 cm^−1^ are attributed to the stretching vibration of CN heterocycles corresponding to the C = N and C–N bonds in the heterocyclic ring. In the synthesized samples, a broad band between 2900 and 3600 cm^−1^ was observed (Kuila et al. 2020; Sarkar et al. 2020; Wei et al. 2023; Zhang et al. 2016a). This band is attributed to the N–H stretching mode, which indicates the presence of NH and/or NH_2_ groups. This broad band is also attributed to O–H and stretching mode. It is known that the O–H stretch tape envelope usually appears between 3500 and 3000 cm^−1^. This band at 3100–3300 cm^−1^ shows the stretching vibration of the O–H bands because of adsorbed water molecules. By adding water molecules to the isolated CN molecule, it is shown by DFT calculations that the vibrational modes in this region increase as the amount of water in the structure increases. The C–N and C = N stretching modes (1232 cm^−1^, 1313 cm^−1^, 1402 cm^−1^, and 1551 cm^−1^) shift to higher frequencies by approximately 4 cm^−1^ in both experimental and simulated IR spectra as increased doping rate of Ho (Kuila et al. 2020).

**Table 3:** Experimental FTIR and calculated IR vibrational modes of CN and HoCN

	Experimental				Computational	
Vibrational assignment	CN	HoCN			CN	HoCN
		0.1	0.2	0.3		
Out of plane bending of -NH_2_	594	592	592	598	580	
Breathing mode	808	808	808	808	818	810
C-N (sp3) stretching	1232	1238	1240	1250	1257	1286
	1313	1315	1315	1322	1336	1379
C-N (sp3) and/or C = N (sp2) stretching	1402	1406	1406	1416	1408	1444
	1452				1466	1466
	1551	1558	1556	1566	1581	1552
	1628	1620	1614	1614	1610	1595
N–H stretching	2900–3600				3482	3482

The raw Raman spectra of CN and HoCN are given in Fig. 4b. In the literature, the Raman peaks of CN have D and G bands, which are around 1350 and 1560 cm^−1^, respectively (Cao et al. 2016; Maślana et al. 2020; Rono et al. 2021; Wang et al. 2015a; Zinin et al. 2009). Both bands overlapped in our Raman spectra and cannot be distinguished. The Raman spectra of the CN exhibited six strong peaks in the range 470 to 1375 cm^−1^. These peaks at 472, 708, 752, and 981 cm^−1^ are related to s-triazine ring breathing modes, and the peaks observed at 1152 and 1237 cm^−1^ are attributed to the aromatic C–N heterocycle stretching vibrations of CN (Cen et al. 2021; Maślana et al. 2020; Mohanraj et al. 2021; Tonda et al. 2014; Zinin et al. 2009).

The Raman spectra of HoCN do not show any additional peak due to doping of Ho. However, as can be seen from Fig. 4b, as the doping ratio increases, the intensity of the peak around 981 cm^−1^ increases compared to the other peaks. This situation is frequently observed in doping studies, and the rate of increase in intensity increases with the doping rate, which means that the doping process has led to an increment in the number of phonon modes that are Raman active (Casiraghi 2009; Kharlamova et al. 2017; Kuila et al. 2020; Li et al. 2023). Upon comparing CN with HoCN samples, a redshift of about 6 cm^−1^ was observed in the characteristic Raman peak at 708 cm^−1^, as seen in Fig. 4b and Table 4. As the doping amount increased, the Raman peak at 708 cm^−1^ shifted to lower wavenumbers, which can be caused by increased length between the carbon and nitrogen atoms. (Joseph and Jemmis 2007; Yang et al. 2013). In support of this situation, the BET results also show that the surface area increased as the doping amount increased, and the calculated bond lengths increased as seen in Table 1.

**Table 4:** Positions of the Raman peaks (cm^−1^) of the CN and HoCN samples

Vibrational Assignment	CN	HoCN		
		0.1	0.2	0.3
S-triazine ring breathing	472	473	474	476
	708	701	702	702
	752	751	752	753
	981	983	983	984
C-N stretching	1152	1152	1153	1155
	1237	1250	1251	1250

#### XPS

**Fig. 5:**
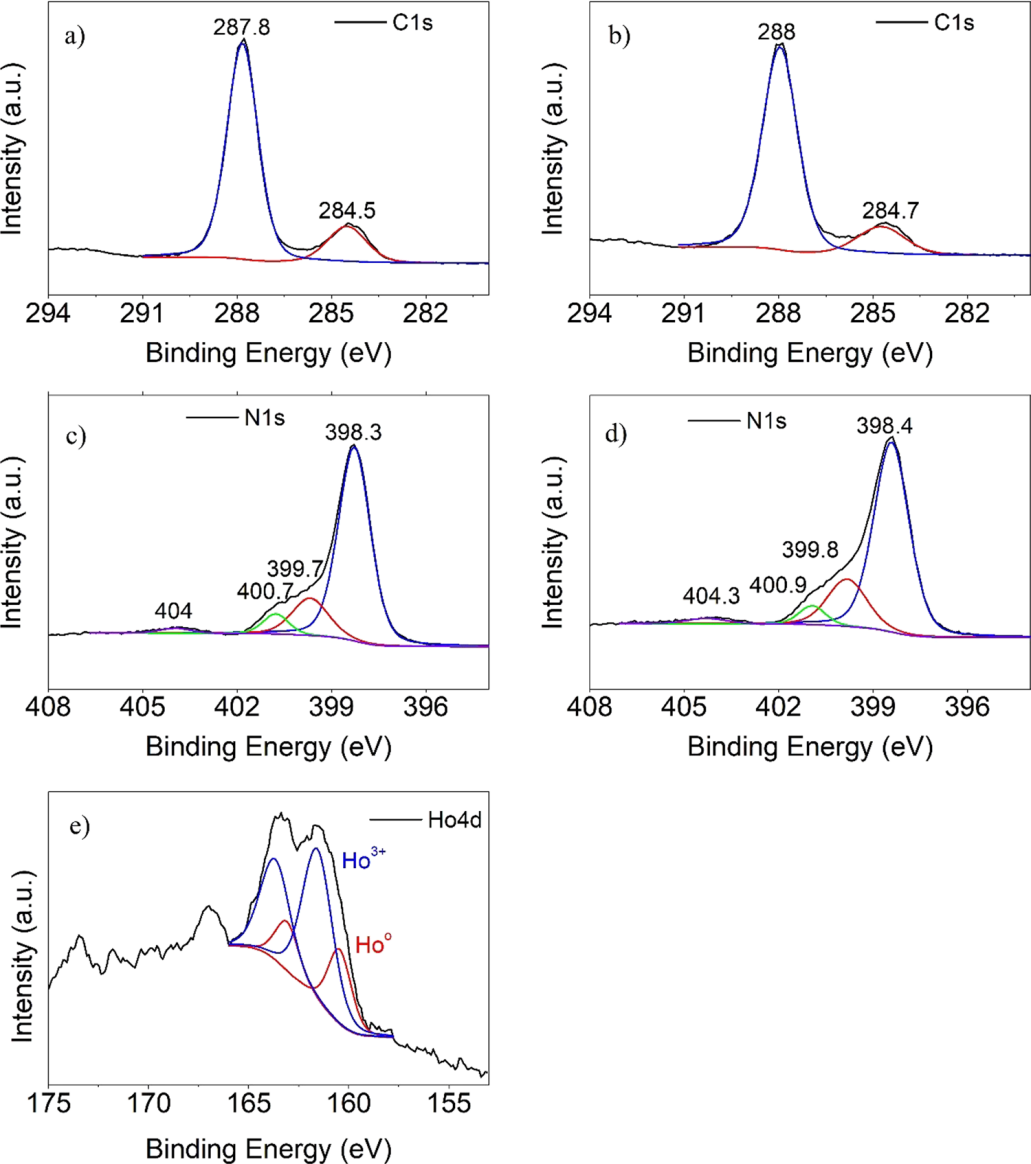
XPS spectra of pristine CN, and HoCN **a**, **c** C1s; **b**, **d** N1s, **e** Ho4d, respectively

X-ray photoelectron spectroscopy (XPS) technique was applied to comprehend the electronic properties, elemental composition, and chemical bond structures of CN and HoCN. Figure 5 and Table 5 show the C1s, N1s, and Ho4d core levels of CN and HoCN compounds. As shown in the figure, C1s core level signal was fit with two peaks; the positions of the peaks originating from N–C = N and C = C in aromatic rings of CN are 287.8 and 284.5 eV, respectively. The N1s core level signal was fit with four distinct peaks with peak positions at 404, 400.7, 399.7, and 398.3 eV, and the source of these peaks are N–H groups, C–N–H, C–N–H, N–C_3_, and C = N–C, respectively (Kuila et al. 2020; Wang et al. 2015a). As can be observed from Fig. 5, the N1s and C1s peaks shifted to higher energies of 0.2 eV on average after Ho doping. As mentioned in the computational results section, after the Ho atom is placed in the center of CN, the N–C bond lengths change as this Ho atom attracts the N atoms in the periphery of the vacancy. This may be the reason why the C1s and N1s binding energies shift to energies as high as 0.2 eV after Ho doping. Also, the absence of an extra peak after Ho doping in both the N1s and C1s signals may be due to the absence of a bond between these atoms and Ho. The XPS spectra of Ho4d signal can be deconvoluted into four peaks, revealing the oxidation states of Ho atoms in the HoCN photocatalysts (Fig. 5). Fitting of Ho4d spectra reveal the presence of two states of Ho, which are metallic Ho^0^ and Ho^3+^ (Ho_2_O_3_) with 160.5 and 161.6 eV binding energies, respectively. The XPS fitting spectra indicate that Ho^3+^ is the most dominant component in HoCN, the ratio of which is higher than that of Ho^0^. As indicated by the Ho4d_5/2_ and Ho4d_3/2_ peaks at 160.5, 163.1, 161.6, and 163.7 eV, for metallic Ho^0^ and Ho^3+^, respectively. (Fan et al. 2021; Osial et al. 2018; Yin 2013).

**Table 5:** Peak parameters of XPS results

CN				HoCN			
Peak	Position	FWHM	%Con	Peak	Position	FWHM	%Con
O1s	532.1	2.5	2.5	O1s	531.8	2.5	3.6
N1s	398.3	1.2	52.9	N1s	398.4	1.3	54.5
N1s	399.7	1.4		N1s	399.8	1.5	
N1s	400.8	0.9		N1s	400.9	1.0	
N1s	404.0	1.2		N1s	404.3	1.5	
C1s	284.5	1.5	47.1	C1s	284.7	1.8	45.4
C1s	287.8	1.2		C1s	288.0	1.3	
				Ho4d_5/2_	160.5	1.4	0.2
				Ho4d_3/2_	163.1	1.4	
				Ho4d_5/2_	161.6	1.8	
				Ho4d_3/2_	163.7	1.8	

#### Optical analysis

**Fig. 6:**
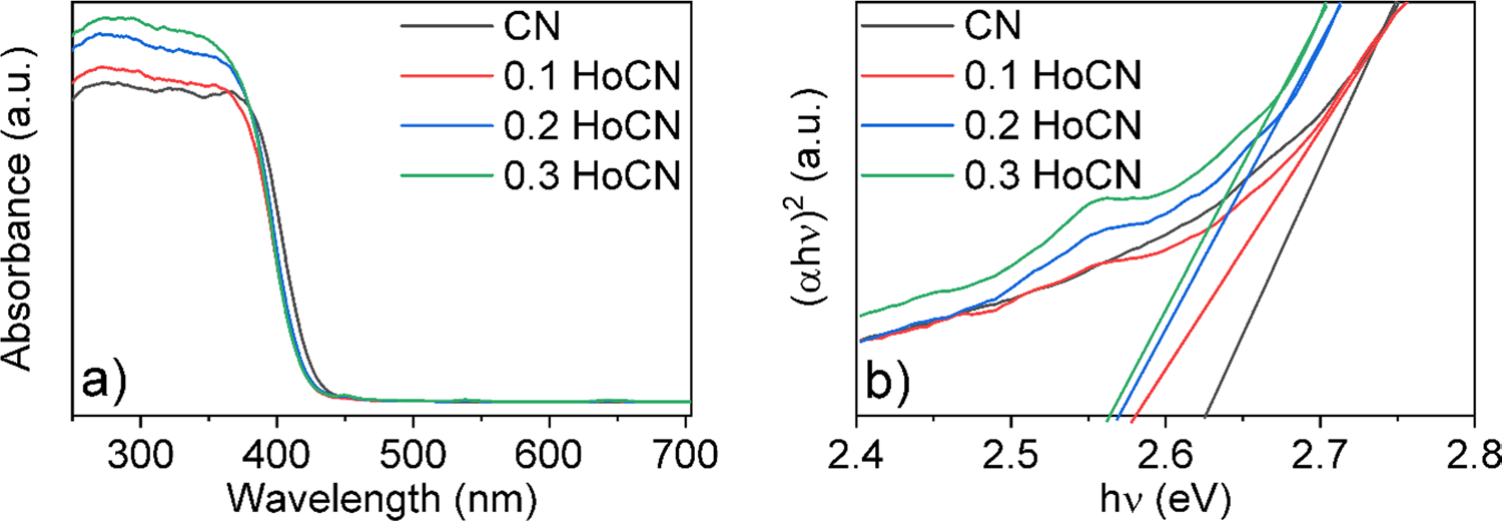
**a** UV–Vis diffuse absorption spectra and **b** Tauc plot of the samples

The absorbance spectra of the synthesized photocatalysts were obtained using diffuse reflectance spectroscopy (DRS). The reflection spectra obtained from DRS were converted to absorption spectra depicted in Fig. 6 by applying the Kubelka–Munk function. As seen in Fig. 6a, all samples can absorb in the visible region (400–700 nm), and a red shift is observed with the doping of holmium which was consistent with the PL results. To calculate the energy band gap of the photocatalysts, Tauc equation was applied and demonstrated in Fig. 6b. By estimating the linear part of the curve to intersect with the *x*-axis, the band gap energy of the photocatalysts can be found. According to this analysis, bandgap energies of the CN, 0.1 HoCN, 0.2 HoCN, and 0.3 HoCN were estimated as 2.62 eV, 2.58 eV, 2.57 eV, and 2.56 eV, respectively. The fact that these values are close to each other means that the holmium doping amount is quite low, and as can be seen, the lowest value is for 0.3 HoCN. According to this analysis, although the photocatalytic activity should be the best for 0.3 HoCN, which has the lowest energy band gap, 0.2 HoCN showed the best result as stated in the conclusion. This can be thought of as a contribution greater than 0.2 blocking the path of light and inability to prevent electron–hole recombination further.

#### TGA

**Fig. 7:**
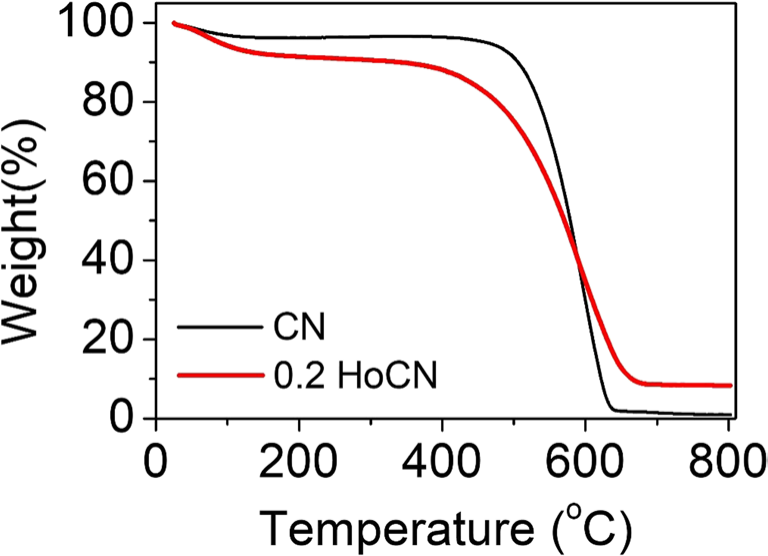
TGA curves of the pure CN and 0.2 HoCN photocatalysts

To discover the thermal stability of the produced samples, changes in the weights of the photocatalysts were observed depending on temperature via thermal gravimetric analysis (TGA). TGAs of the pure and 0.2 HoCN photocatalysts which are given in Fig. 7 were performed under a nitrogen atmosphere between 25 and 800 °C at a heating rate of 10 °C/min. For both samples, small rates of weight loss can be found in the temperature range of 25–170 °C, corresponding to the loss of coordinated water molecules. Both samples remain thermally stable when the temperature reaches around 450 °C. While the decomposition rate of CN is quite high in the range of 500–650 °C, the decomposition rate of the holmium-doped one is slower. It is clearly seen that Ho doping slows down the decomposition rate of CN in the range of 500–700 °C. In the final case at 800 °C, while the Holmium-doped one retained 8.3% of its mass (91.7% mass loss), only 0.97% of the undoped one remained (99.03% mass loss). These results show that while CN completely decomposes at temperatures above 700 °C, holmium doping reduces decomposition by leaving residue behind.

#### Morphological analysis

**Fig. 8:**
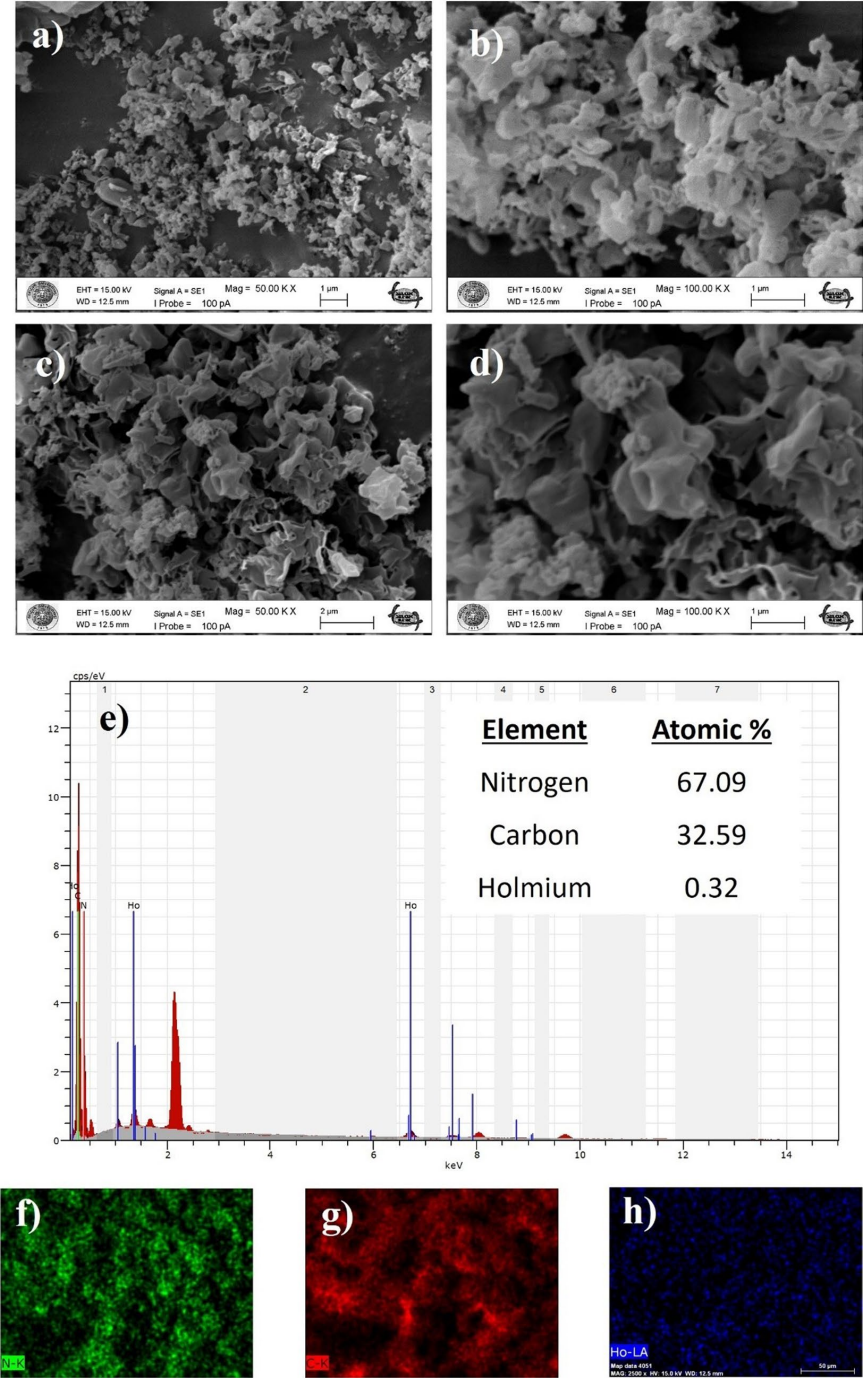
SEM images of the pure (**a**, **b**) and 0.2 HoCN nanoparticles (**c**, **d**), and EDX analysis of 0.2 HoCN (**e**) and elemental mapping of N (green), C (red), and Ho (blue) (**f**, **g**, **h**)

Figure 8 shows the SEM images of the pure and 0.2 HoCN nanoparticles at different magnifications. While Fig. 8a and b show 50 K and 100 K magnifications of the pure CN, Fig. 8c and d show 50 K and 100 K magnifications of the doped one. As seen from the figures, the porous and wrinkled structure of CN spreads like a sheet with the addition of holmium, and the nanoparticles exhibit a nanosheet-like structure. This nanosheet structure can be attributed to increased specific surface area and active sites with the addition of holmium (Xia et al. 2022). These results are in good agreement with our BET results which are depicted in Fig. 10. In addition, energy dispersive X-ray (EDX) analysis of 0.2 HoCN nanoparticles divulges the presence of holmium, carbon, and nitrogen in the nanoparticles, indicating that holmium dopant is in the desired ratio. Elemental mapping images of 0.2 HoCN which are given in Fig. 8f, g, h confirm the EDX analysis and demonstrate the uniform distribution of N (green), C (red), and Ho (blue).

#### PL

**Fig. 9:**
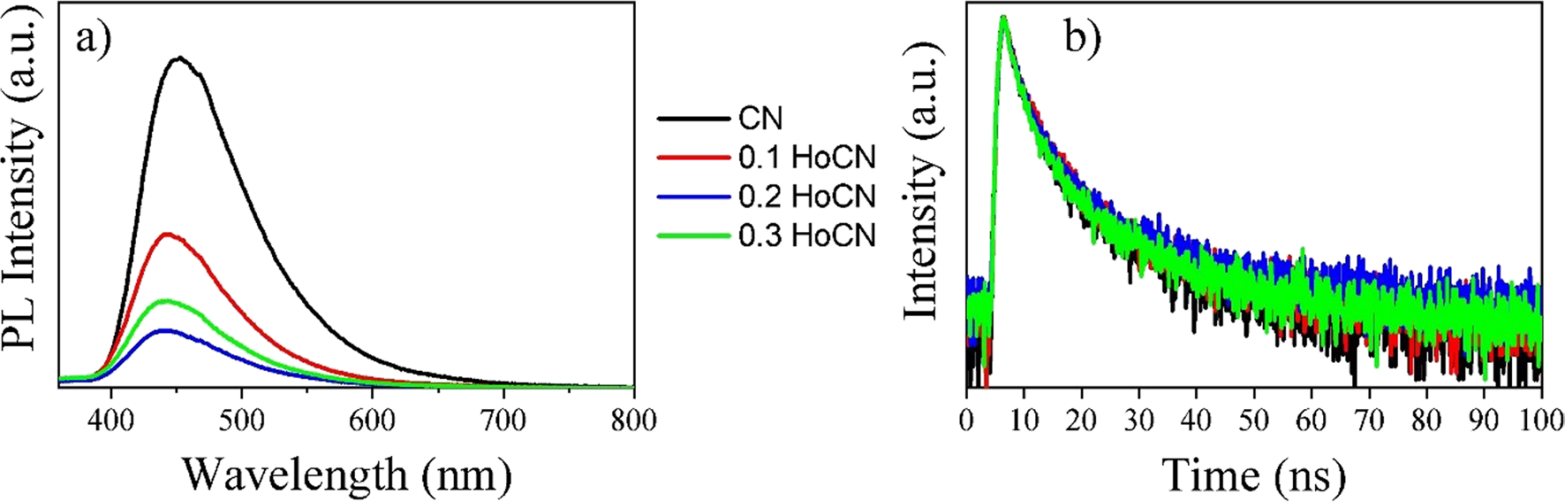
PL spectra (**a**) and TRPL decay curves (**b**) of the pure and Ho-doped CN samples

To further examine the effect of holmium doping on electron–hole separation, the photoluminescence (PL) spectra of the specimens were examined with 330-nm wavelength excitation at room temperature. As seen in Fig. 9a, the emission intensities of all samples are in the range of 400–600 nm. It is noticeable that the peaks of the curves of the doped samples are lower than that of the pure ones, which is attributed to the reduced electron–hole recombination rates. Moreover, when the peak values of the doped samples are compared with each other, it is seen that the value of the 0.2 doped sample is lower than the others, which is favorable for its photocatalytic performance (Li et al. 2020). Figure 9 b shows TRPL decay curves of all samples, and Table 6 shows lifetime of photogenerated carriers. As seen in the table, average lifetime (*τ*_*a*_) of 0.2 HoCN is longer than others. As can be seen from Table 6, as the doping ratio increases, *τ*_*a*_ increases, while it decreases dramatically at 0.3 HoCN. This is also consistent with PL signals. A much quenched PL signal and a longer lifetime indicate that doping Ho could effectively suppress charge recombination rate and enhance photocatalytic activity.

**Table 6:** Lifetime of photogenerated carriers

Sample	*τ*_1_ (ns)	*τ*_1_ (ns)	*τ*_*a*_ (ns)
CN	2.93	15.68	9.49
0.1 HoCN	3.13	15.29	10.02
0.2 HoCN	3.04	16.38	10.27
0.3 HoCN	2.68	14.52	9.33

#### BET analysis

**Fig. 10:**
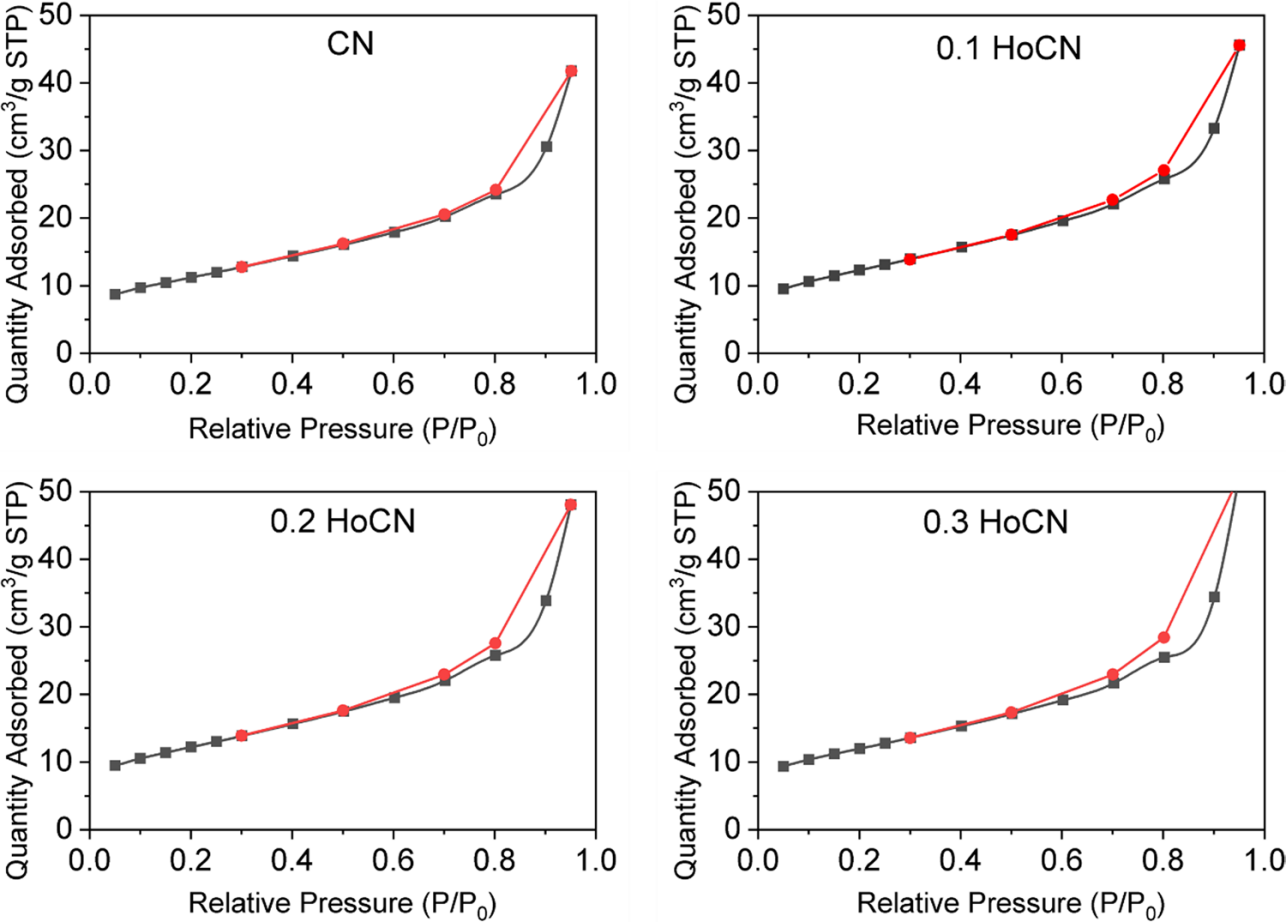
N_2_ adsorption–desorption isotherms of the pure and doped samples

Textural properties such as specific surface areas and pore widths of the pure and HoCN samples were determined through nitrogen adsorption–desorption data of the pure and doped samples. Figure 10 shows the nitrogen adsorption–desorption isotherms of the pure and doped samples obtained by Brunauer–Emmett–Teller (BET) analysis at 77 K. The results illustrate that all samples have type IV mesoporous structures with the hysteresis loop of H3 type in the range of 0.5–0.94 of the relative pressure (*p*/*p*_0_) (Ke et al. 2014). Specific surface areas and average pore widths of the photocatalysts are given in Table 7. Although doping has caused an increment in the absorption surface area for all samples, no distinctive change was observed depending on the doping amount. Results also indicate that average pore widths vary between 6 and 7.8 nm. Compared with the pure one, the increase in the values after %0.2 doping is remarkable. Generally, the textural properties, such as specific surface area and width of the obtained mesoporous samples, have changed with Ho modification, which has improved the photocatalytic activity.

**Table 7:** Textural properties of the pure and doped samples

Samples	Bet Surface Area (m^2^/g)	Average Pore Size (nm)
CN	39.4034	6.559
0.1 HoCN	43.1135	6.542
0.2 HoCN	42.8439	6.943
0.3 HoCN	41.9018	7.731

### Photocatalytic performance of the photocatalysts

**Fig. 11:**
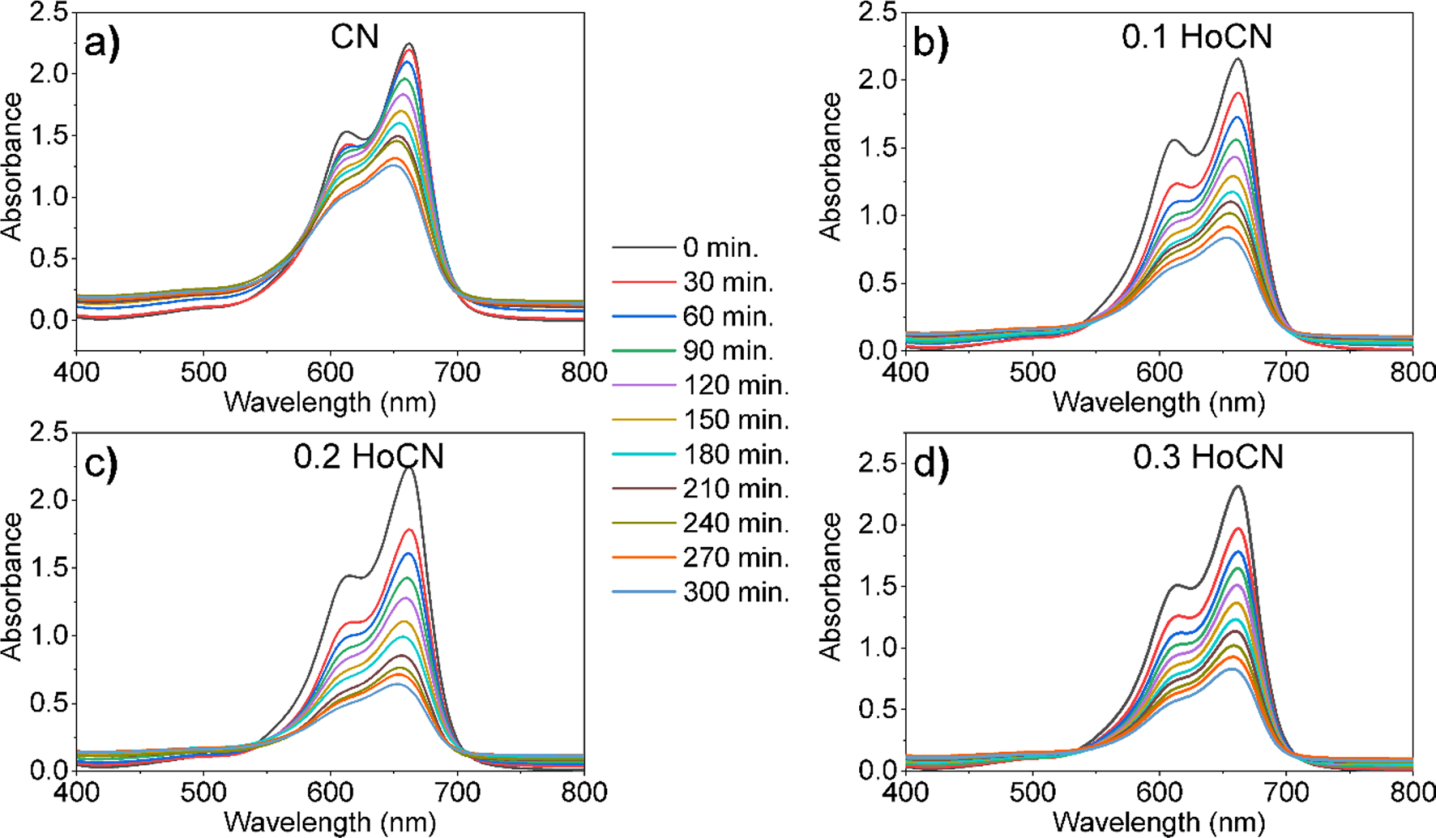
Absorbance spectra of MB dye solutions over time with added **a** the pure, **b** 0.1 HoCN, **c** 0.2 HoCN and **d** 0.3 HoCN photocatalysts

To comprehend the ascendancy of the holmium dopant ratio on the photoactivity of the CN, the photocatalytic activities of the pure CN and different amounts of Ho-doped CN photocatalysts have been evaluated by degradation of MB dye under visible light irradiation. As seen in Fig. 11, experiments were performed with the photocatalysts and MB under visible light irradiation with a visible exposure time of 300 min. The wavelength of maximum absorbance of MB at 662 nm was used for the measurement of MB concentration. No changes in MB absorption peak position were observed during the degradations in Fig. 11. This shows that de-ethylation is assigned to the shift of the peaks and is not observed during the degradation of MB in the existence of photocatalysts.

**Fig. 12:**
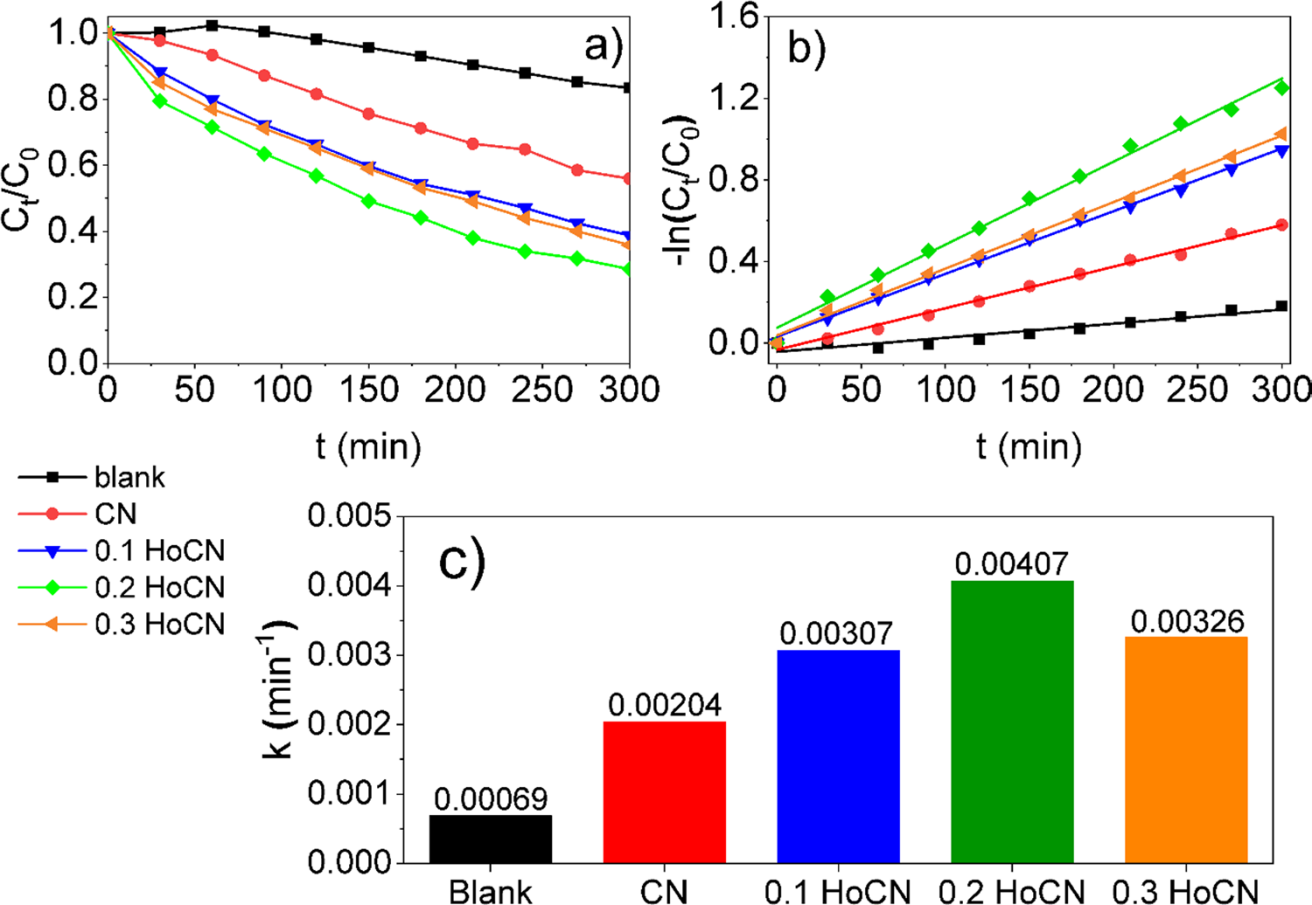
**a** Change of the concentrations of MB solution in the presence of the samples and blank under visible irradiation. **b** Kinetics of MB dye solutions. **c** Column graph of the kinetic values

Change of concentrations of MB solution with or without samples under visible light are given in Fig. 12a. MB concentration depicted as blank in Fig. 12a has remained generally stable in the absence of the photocatalysts. The pure CN has the lowest degradation under visible light illumination, and the degradation rate of MB for the pure CN is about 44%. The degradation of MB changes with the doping of CN with Ho doping. The content of Ho doping is an important quantity on the CN photocatalysis. It is seen that the degradation rate which is calculated from the direct relationship between concentration and absorbance (Hu et al. 2021) reaches an optimum value under visible irradiation (71.4%) at 0.2 Ho doping. Doping Ho more than 0.2 reduces the degradation of MB and worsens the photocatalytic activity of CN. According to this result, kinetic value of 0.2 HoCN photocatalyst is two times higher than the value of the pure one. This result is compatible with the study performed with Eu-doped CN (Xu et al. 2013a) and higher than the results obtained for Ce-doped CN (Kuila et al. 2020) in the literature which were performed with MB dye under the visible light.

It is known that the Langmuir–Hinshelwood (L–H) model scrutinizes the chemical kinetic of a degradation process. In the L–H model, degradation can be described using a first-order reaction that goes ahead at a rate depending linearly on the concentration. If $k$, ${C}_{0}$, and ${C}_{t}$ are defined as a first-order rate constant, first concentration, and final concentration, respectively, the L–H model reduces to the following equation (Karacaoglu et al. 2023):

7 $$-\mathit{ln}\frac{{C}_{t}}{{C}_{0}}=kt$$

The plots of $-\text{ln}\left({C}_{t}/{C}_{0}\right)$ versus time *t* are presented in Fig. 12b to calculate the kinetic values of the photocatalytic reactions which were given as a column graph in Fig. 12c. According to the results obtained, 0.2 HoCN nanoparticles have the highest $k$ value which is 0.004 min^−1^, and this result is more than two times greater than the undoped CN.

**Fig. 13:**
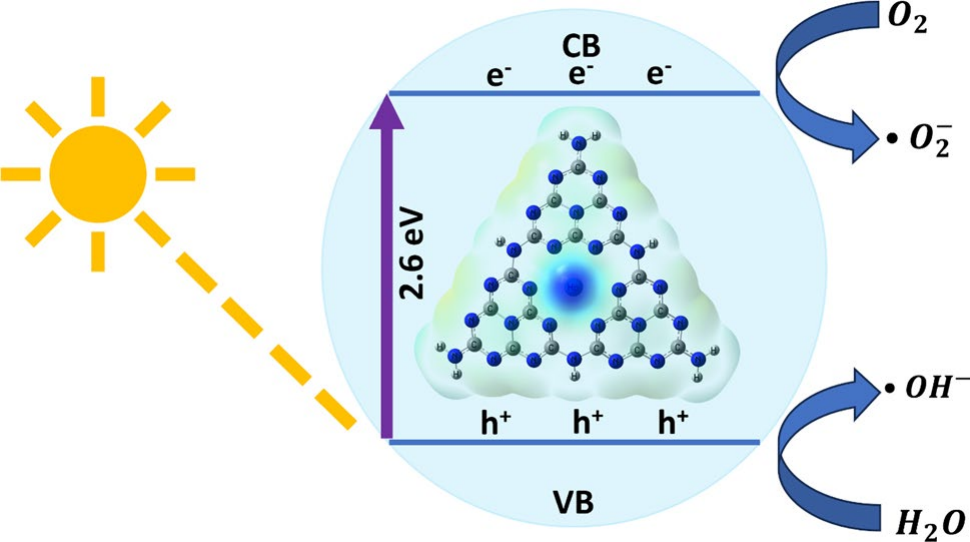
Photocatalytic mechanism of holmium-doped CN photocatalysts

An explanation of the photocatalytic mechanism of holmium-doped CN under visible light is schematically illustrated in Fig. 13. By applying photons with energies greater than the energy band gap to photocatalysts, electrons in the valence band are excited towards the conduction band, leaving holes behind. Photogenerated electron–hole pairs enable the formation of reactive oxygen species in the aqueous solution of methylene blue. While photogenerated electrons react with dissolved oxygen in the solution to form superoxide anions ($\bullet {\text{O}}_{2}^{-}$), photogenerated holes combine with water molecules to form hydroxyl radicals (•${\text{OH}}^{-}$):

8 $$\text{HoCN}+h\nu \to \text{HoCN }\left({\text{h}}^{+}+{\text{e}}^{-}\right)$$

9 $${\text{O}}_{2}+{\text{e}}^{-} \to \bullet {\text{O}}_{2}^{-}$$

10 $${\text{h}}^{+}+{\text{H}}_{2}\text{O }\to {\text{H}}^{+}+\bullet {\text{OH}}^{-}$$

These reactive oxygen species play a leading role in the degradation of the dye by breaking down MB molecules. Another important role in visible light photocatalysis belongs to the energy bandgap of CN. The impurity energy levels resulting from holmium doping cause narrower band gaps than the pure CN. Narrowing of the band gap increases visible light absorption and leads to an increase in photocatalytic activity. The umbrella-like structure of HoCN shown in Fig. 1 has geometrically more surface area. Consistent with the BET results, the higher surface area of HoCN offers more photocatalytic active centers than pure CN. Furthermore, electrons from the valence band of CN can be kept in the 4f energy levels of these elements. As a result, holmium ions settling on CN form oxygen vacancies and surface defects, which enable dye adsorption and effective separation of electron–hole pairs. Oxygen vacancies bond with electrons to create an excited energy level lower than the conduction band of the CN, allowing effective visible light absorption. In this manner, HoCN nanoparticles exhibit better photocatalytic activity than the pure ones. However, as seen in Fig. 12c, Ho doping increases the photocatalytic activity of the CN nanoparticles up to a critical level. Our study determines this critical level in the nanoparticles as 0.2 Ho by weight. Doping more than this amount blocks the path of light thereby photocatalytic activity of the nanoparticles will decrease.

## Conclusion

In this work, the effect of Ho modification on the determination of the photocatalytic efficiency of CN was analyzed in detail, both theoretically and experimentally. Comprehensive analysis of theoretical calculations has shown that Ho modification ensures the CN molecule is a more chemically stable and effective sorbent, which will significantly affect the performance of the material under visible light. Moreover, the optimum doping amount to provide maximum photocatalytic efficiency was determined experimentally and theoretically by gradually changing the molarity of holmium from 0.1 to 0.3 mmol. Raman, BET, XRD, and SEM measurement results are compatible with each other and show that all samples have porous nanostructure and some properties such as bond length in some intervals and absorption surface areas ascent in direct proportion to the amount of doping. Δ*E* gap values obtained via absorbance measurement are smaller than those obtained for the pure sample. Unlikely this behavior, it was observed from the PL measurements that the peaks of the curves show a decreasing tendency up to 0.2 mmol doping, then start to increase.

Generally, one can conclude that Ho doping is a successful strategy to overcome challenges such as high recombination rate of electron–hole pairs, low surface-active area, and poor electrical conductivity. Although the improvement in the properties of g-C3N4, such as the surface active area, cannot be distinguished precisely depending on the doping ratio, the increase in *k* values of the photocatalytic reactions was remarkable when compared to that of the pure sample. It was also clearly evident that the photocatalytic performances of CN samples doped with 0.1 and 0.3 mmol Ho were very close to each other, and also both were lower than the 0.2 mmol doped sample. This can be ascribed to the fact that electron–hole formation could be the dominant process in determining the optimum doping value and maximum photocatalytic performance. In particular, among all the doped samples, it is very striking that the photocatalytic efficiency of 0.2 mmol doped sample is two times higher than the pure one, which is very promising for further applications.

## Data Availability

Data will be made available on reasonable request.
